# Benchmarking bioinformatic virus identification tools using real-world metagenomic data across biomes

**DOI:** 10.1186/s13059-024-03236-4

**Published:** 2024-04-15

**Authors:** Ling-Yi Wu, Yasas Wijesekara, Gonçalo J. Piedade, Nikolaos Pappas, Corina P. D. Brussaard, Bas E. Dutilh

**Affiliations:** 1 Theoretical Biology and Bioinformatics, Science4Life, Utrecht University, Padualaan 8, Utrecht, 3584 CH The Netherlands; 2 Institute of Bioinformatics, University Medicine Greifswald, Felix Hausdorff Str. 8, 17475 Greifswald, Germany; 3 Department Marine Microbiology and Biogeochemistry, NIOZ Royal Netherlands Institute for Sea Research, Den Burg, PO Box 59, Texel, 1790 AB The Netherlands; 4 Institute for Biodiversity and Ecosystem Dynamics, University of Amsterdam, Amsterdam, The Netherlands; 5 Institute of Biodiversity, Faculty of Biological Sciences, Cluster of Excellence Balance of the Microverse, Friedrich Schiller University Jena, 07743 Jena, Germany

## Abstract

**Background:**

As most viruses remain uncultivated, metagenomics is currently the main method for virus discovery. Detecting viruses in metagenomic data is not trivial. In the past few years, many bioinformatic virus identification tools have been developed for this task, making it challenging to choose the right tools, parameters, and cutoffs. As all these tools measure different biological signals, and use different algorithms and training and reference databases, it is imperative to conduct an independent benchmarking to give users objective guidance.

**Results:**

We compare the performance of nine state-of-the-art virus identification tools in thirteen modes on eight paired viral and microbial datasets from three distinct biomes, including a new complex dataset from Antarctic coastal waters. The tools have highly variable true positive rates (0–97%) and false positive rates (0–30%). PPR-Meta best distinguishes viral from microbial contigs, followed by DeepVirFinder, VirSorter2, and VIBRANT. Different tools identify different subsets of the benchmarking data and all tools, except for Sourmash, find unique viral contigs. Performance of tools improved with adjusted parameter cutoffs, indicating that adjustment of parameter cutoffs before usage should be considered.

**Conclusions:**

Together, our independent benchmarking facilitates selecting choices of bioinformatic virus identification tools and gives suggestions for parameter adjustments to viromics researchers.

**Supplementary Information:**

The online version contains supplementary material available at 10.1186/s13059-024-03236-4.

## Background

Viruses of microbes (VoMs) are the most abundant life entities on Earth [1–3], infecting many microbes at any given time [4]. The interactions between VoMs and their microbial hosts can change microbial host community composition [5] and their physiology [6], affecting not only the health of higher organisms (including plants, animals, and humans) [7–9] but also the global biogeochemical processes [10, 11]. Besides the direct (killing host) and indirect (e.g., recycling of limiting nutrients) ecological effects of viral lysis, VoMs can influence microbial hosts’ physiology by altering metabolic pathways through horizontal gene transfer or through the expression of metabolic genes carried within viral genomes during viral infection [6, 12, 13]. Understanding the complex interactions between VoMs and their microbial hosts and the ecological roles of VoMs could contribute insights on solving crucial global problems, such as infectious diseases, climate change, and food crisis [14–16]. Still, improved knowledge on the genetic diversity of VoMs is warranted [17–19]. Acknowledging the genetic diversity of VoMs and their high potential for application, there is considerable interest in “mining” these viral sequences to discover new candidates for antimicrobial drugs, enzymes for biotechnology, and bioremediation purposes [20–22].

Originally, the discovery of most viruses depended primarily on the observation of their effect on a host. Nowadays, (meta)genomics has become a major method for virus discovery. Despite the fact that there is no universal marker gene carried by all viruses, studies of specific hallmark genes have revealed large-scale viral diversity of certain viral groups from marine and host associated biomes [23–28]. For example, Sakowski et al. 2014 and Wu et al. 2023 found great novel dsDNA bacteriophage diversity from marine samples using ribonucleotide reductase [23, 28], while Wolf et al. 2020, Zayed et al. 2022, and Edgar et al. 2022 expanded the known RNA viruses using RNA-dependent RNA polymerase as a marker [25–27]. Complementary to marker gene approaches, shotgun metagenomic sequencing is suitable for discovering viruses without targeted marker genes [29, 30]. Through the shotgun metagenomic approach, total genetic material is extracted from environmental samples, either from an enriched viral fraction (virome) or from a fraction representing both viruses and their hosts (total metagenome). This is then sequenced randomly. It is common for viral reads to comprise less than 5% of metagenomic sequences from a total metagenome sample [29]. Viromic datasets are derived from samples that were enriched for viruses by, e.g., size filtration, density gradient centrifugation, and/or chemical concentration [15, 31, 32]. Notwithstanding advances in virus filtration techniques [29, 31, 33], it can still be challenging to distinguish viruses in metagenomic/viromic datasets due to (1) the lack of viral marker genes [23], (2) limited availability of viral reference genomes [15], (3) the possible high sequence similarity between viral and microbial genomes [34], and (4) the presence of prophages in microbial genomes. Therefore, it has become of great interest to determine which sequences within whole microbial communities are derived from viruses. These sequences can occur as free virions, active intracellular infections, particle or host-attached virions [35], and host-integrated or episomal viral genomes (i.e., proviruses) [29, 30, 36].

In the past years, many bioinformatic virus identification tools have been developed for the task of identifying viral sequences in mixed metagenomic datasets (Additional file 2: Table S1). Some of the tools rely strongly on comparing candidate sequences to the sequences in reference databases. For example, VirSorter [37] uses information on the enrichment in viral-like genes, depletion in Pfam-affiliated genes, enrichment in short genes, and depletion in strand switching. MetaPhinder [38] uses BLASTn and average nucleotide identity thresholds to classify viral contigs in metagenomes. Sourmash [39] employs MinHash-based algorithms to quickly compare large sets of sequences and estimate their similarity to viruses. Some of the tools used machine learning techniques that also detect other genomic features based on positive and negative training sets. Seeker employs long short-term memory (LSTM) models that can identify distant dependencies within sequences, to distinguish phages from bacteria. VirFinder [40] is a logistic regression classifier using nucleotide sequence 8-mers as features to identify viral sequences. Some of the machine learning based tools use convolutional neural networks (CNNs). DeepVirFinder [41] and PPR-Meta [42] use CNNs that encode long- and short-range features of viral genomes. Some of the machine learning tools use hybrid approaches to combine the advantage of homology search and machine learning. VIBRANT [43] uses viral nucleotide domain abundances in a neural network framework to classify contigs having more than four proteins. VirSorter2 [44] integrates the biological signals of VirSorter in a tree-based machine learning framework. In addition, the training/reference databases used in virus discovery are diverse, including sequence databases such as RefSeq [45], virome datasets in specific studies [37, 41, 44], and HMMs [46] of pVOGs [43, 47]. The number and diversity of virus identification tools make it challenging to choose the right tool(s), parameters, and cutoffs.

Several benchmarking studies have compared the performance of various virus identification tools [48–53] (Additional file 2: Table S2). Most of them used simulated sequencing data or sequencing data from mock community as testing datasets. The known fraction of the viral sequence space is limited because most viruses remain uncharacterized. To perform reliable benchmarking, one would have to set aside a fraction of the small collection of known virus sequences before using the remainder as training or reference data for the tools. As many virus identification tools included RefSeq data in their training/reference databases, using RefSeq to generate a mock sample may be expected to bias the results (more true positives). Besides, to avoid overlap between training and testing data, one would have to address the redundancy in the RefSeq database which contains many similar cultivated and isolated viral genomes while viruses in environmental samples are diverse and remain under-characterized. Finally, both the macro- and micro-diversity of viruses in natural samples is usually a lot greater than the diversity of viruses in the database, further obscuring the benchmarking results. Ho et al. [53] benchmarked tools on a previously sequenced mock community containing five phage strains, which is low compared to real environmental samples. The newest benchmarking from Schackart et al. [52] included two real-world metagenomic datasets but did not define a ground truth dataset.

**Fig. 1:**
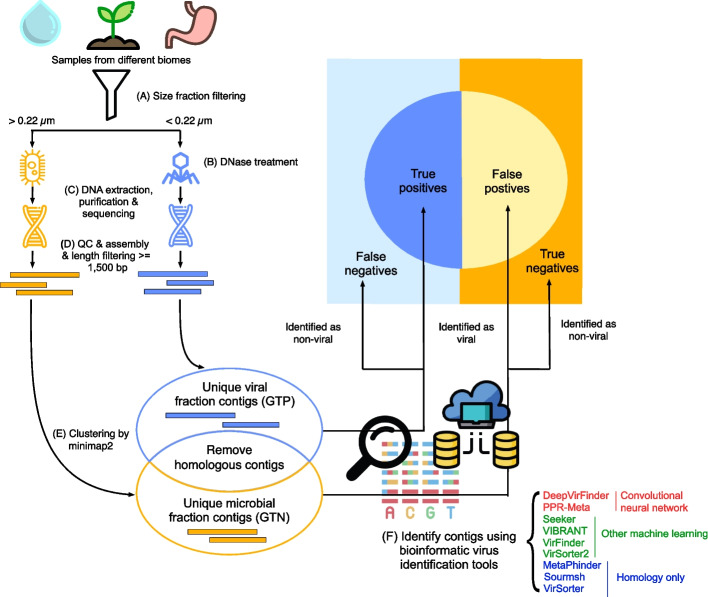
Real-world metagenomic data benchmarking pipeline. **A** Samples from three different biomes were size fraction filtered through 0.22 μm filters to obtain microbial- (> 0.22 μm) and viral-enriched fractions (< 0.22 μm). **B** DNase treatment was performed in viral-enriched fractions to remove free DNA before viral lysis. **C** DNA was separately extracted, purified, and sequenced from microbial- and viral-enriched fractions to obtain viral and microbial datasets. **D** Sequenced DNA reads were quality-controlled and assembled into longer contigs. Contigs with lengths shorter than 1500 bp were excluded from downstream analysis. **E** Homologous contigs between viral and microbial datasets were found using minimap2 and removed. Unique viral fraction contigs and unique microbial fraction contigs were used as ground truth positives and negatives, respectively. **F** Nine bioinformatic virus identification tools were applied to these datasets. Tool names are colored based on algorithms: convolutional neural network tools (red), other machine learning tools (green), and homology-only tools (blue). Viral contigs that are identified as viral and non-viral are considered as true positives and false negatives, respectively. Microbial contigs that are identified as viral and non-viral are false positives and true negatives, respectively

To avoid biases in our comparison and reach the complexity level of microbial/viral communities in real-world metagenomic datasets, we used simulated and real-world metagenomic data as testing datasets. We benchmarked nine state-of-the-art bioinformatic virus identification tools on paired viral and microbial samples across three vastly distinct biomes, including seawater (this study) [54], agricultural soil [31], and human gut [55] (Additional file 2: Table S3 and S4). These three representative biomes were distinct in microbial community compositions and diversity [56], and we anticipate that viral community would follow similar. The selected viral and microbial samples were obtained through physical size fractionation, where samples went through filters with pore size of 0.22 μm to obtain viral (< 0.22 μm) and microbial (> 0.22 μm) fractions [57]. To ensure the quality of the fractioned real-world testing datasets, we (1) selected studies that treated their virome with DNase, (2) assessed the virome quality using ViromeQC, (3) removed the homologous contigs present in both viral and microbial datasets, and (4) validated the viral and microbial fraction contigs using robust bioinformatic tools (Fig. 1 and Additional file 1: Fig. S1).

We collected Illumina sequencing datasets of eight paired viral and microbial samples from each of the three biomes. First, we benchmarked the performance of tools on their default cutoffs. Second, we tested the effect of different parameters and cutoffs on the annotations. Third, we further validated our benchmarking results with extra bioinformatics tools. Our comprehensive analysis of virus identification tools in diverse biomes highlights the trade-off between specificity and sensitivity and provides valuable insights for researchers looking to identify viruses in metagenomic data (Fig. 1 and Additional file 1: Fig. S1).

## Results

We benchmarked nine virus identification tools in thirteen modes using simulated and real-world metagenomic datasets. For the simulated datasets, we generated mock contigs from reference genomes of viruses and bacteria deposited in the RefSeq database after the last tool training database was created. For the real-world metagenomic datasets, we used eight dataset pairs from each of three distinct biomes: seawater, soil, and human gut. Ground truth positives and negatives were defined as metagenomic contigs from viral (< 0.22 μm) and microbial (> 0.22 μm) size filters; overlapping sequences were excluded (Fig. 1 and Additional file 1: Fig. S1).

### Quality and composition of the real-world testing datasets from three biomes

To assess the viral enrichment and microbial contamination level of the viral datasets, we applied ViromeQC. The total enrichment score of the seawater dataset was 65 times higher than the soil dataset and 160 times higher than the gut dataset (Additional file 2: Table S5).

**Fig. 2:**
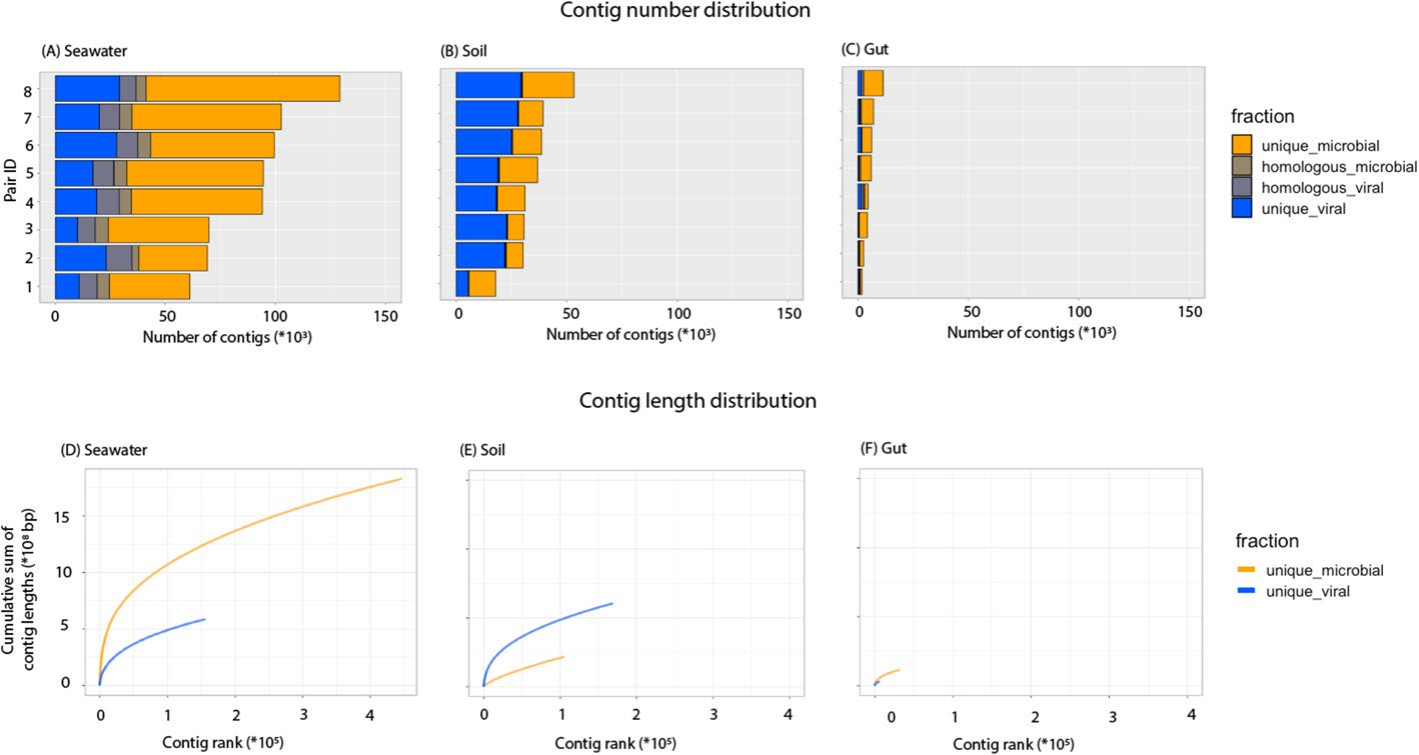
The number of contigs assembled from eight paired samples from seawater (**A**), soil (**B**), and gut (**C**) biomes and their cumulative lengths (**D**, **E**, **F**). *X* and *Y* axes of panels (**A**, **B**, **C** and **D**, **E**, **F**) are scaled to the same maximum values, so the numbers are comparable between biomes

Raw sequencing reads were quality-controlled and assembled into contigs. The assembly statistics, including the contig number and length distributions, are summarized in Fig. 2, Additional file 1: Fig. S2, and Additional file 2: Table S6. In total, seawater datasets contained the greatest number of contigs while gut datasets contained the smallest number of contigs. Seawater and gut datasets contained more microbial contigs than viral contigs while soil datasets contained more viral contigs than microbial contigs and with a greater total length. The soil datasets had the lowest percentage of homologous contigs between the viral and microbial datasets. As overlapping viral and microbial sequences might represent active and integrated temperate viruses, respectively, we hypothesize that there might be more lytic viruses in soil than in seawater and gut, in line with previous findings that lysogeny genes are scarce in soil viromes [11, 58–61]. These overlapping sequences were removed from our benchmarking analysis. Detailed information about the homologous contigs is deposited into the Zenodo repository (seawater_minimap_wtp_output.txt, soil_minimap_wtp_output.txt, and gut_minimap_wtp_output.txt).

Assembly coverage and quality were assessed by re-mapping sequencing reads to the length-filtered assembled contigs per dataset (Additional file 2: Table S7). The percentage of properly paired reads from bwa mem mapping ranged from 11% (gut microbial dataset SRR5665119) to 94% (gut viral dataset SRR5665153) with an average of 52% (23.69%) for all datasets. Viral datasets usually had a greater percentage of properly paired reads than microbial datasets across the three biomes. This might be due to factors such as (1) the larger genomes of microbes, (2) the higher local microbial community complexity, and (3) the persistence of DNA in dead microbial matter [62, 63], which should be removed during the DNase step in the virome preparation but may persist in the microbiomes. Seawater datasets had the best assembly quality with mean percentages of properly paired reads of 79% and 45% for viral and microbial datasets, respectively. The DNA sequences of assembled scaffolds with lengths of at least 1500 bp are deposited into the Zenodo repository (seawater_scaffolds_gt1500.fasta, soil_scaffolds_gt1500.fasta, and gut_scaffolds_gt1500.fasta). We propose that these datasets may be used in future benchmarking studies and that the results may be compared with those presented below.

### Machine learning tools outperformed homology-only tools

**Fig. 3:**
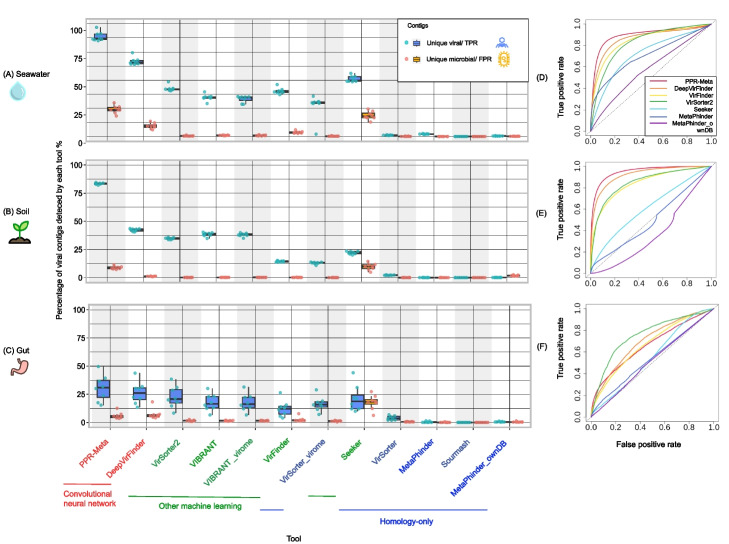
Percentage of contigs identified as viral in the viral (true positive rate, blue) and microbial (false positive rate, orange) datasets, sorted by the average difference in detection rate between eight viral and microbial paired datasets in seawater (**A**), soil (**B**), and gut (**C**) biomes. Colors of the tool names as in Fig. 1. Convolutional neural network tools outperformed other machine learning tools, while the homology-only tools performed the worst. Receiver operating characteristic (ROC) curves of seven methods based on their virus identification scores in seawater (**D**), soil (**E**), and gut biomes (**F**). The dashed diagonal line represents classification according to random chance. The area under the ROC curve (AUC) of each tool is listed in Additional file 2: Table S9

To compare the ability of the tools to detect viral sequences, the true positive rate (TPR, also known as sensitivity) and false positive rate (FPR) were measured based on the percentage of contigs from the viral and microbial size fractions that were identified with default thresholds (Fig. 3A, B, C and Additional file 2: Table S8). Detailed results per contig are available in the Zenodo repository (seawater_minimap_wtp_output.txt, soil_minimap_wtp_output.txt, and gut_minimap_wtp_output.txt). All tools, except for Sourmash, detected viral contigs. Most tools ranked consistently high or low in each of the three biomes. Not many virome contigs were detected in the human gut samples by any tool. This was probably due to the contamination of microbes in the virome datasets (Additional file 2: Table S5). Thus, optimization of experimental protocols to obtain virome from gut/fecal samples is suggested.

The choices of the algorithms and the parameters, the detected biological signals, and the compositions of the training databases all play a role in the ability of the tools to distinguish viral and microbial sequences. CNN tools outperformed other machine learning tools, while the homology-only tools performed the worst. Top performing tools, PPR-Meta (TPR or sensitivity: 68% ± 28%; FPR: 13% ± 8%) and DeepVirFinder (TPR or sensitivity: 45% ± 18%; FPR: 6% ± 5%), use convolutional neural network (CNN) algorithms that can capture short- and long-range signals on viral and microbial genomes. PPR-Meta’s CNN contains two paths—a “base path” and a “codon path” while DeepVirFinder only contains the “base path.” The “base path” is beneficial to extracting the sequence features of coding or non-coding regions while the “codon path” is specifically designed to capture the properties of coding regions. The thorough detection of biological signals encoded in both DNA and codon sequences might contribute to the good performance of PPR-Meta. PPR-Meta also detected many sequences among the microbial contigs, especially from the seawater biome. These could include microbial sequences that were spuriously detected as viral, reflecting a tradeoff between sensitivity and specificity, but it could also be that the microbial fraction still contained viral elements, such as integrated prophages and intracellular viruses in a successful or unsuccessful infection cycle and thus present in the microbial fraction. There were relatively many such matching contigs in seawater samples (Fig. 2), possibly indicating the prevalence of actively infecting viruses in that biome. The viral signals found in the microbial fraction also confirmed the presence of possible viral elements in the microbial fraction (Fig. 5B). We will discuss this further in the below section of “Validation of predicted viral contigs in the real-world testing dataset.” Both PPR-Meta and DeepVirFinder used RefSeq viral genomes as training databases, and DeepVirFinder additionally included virome datasets from specific studies in the training database. Still, DeepVirFinder did not perform better than PPR-Meta in our benchmark.

VirSorter2 and VIBRANT performed similarly, identifying just under half of the viral contigs in seawater and soil metagenomes with few false positives (Fig. 3A, B, C). The two tools use similar biological signals but different algorithms. Both VirSorter2 and VIBRANT use viral domain abundance information as biological signals for identifying viral contigs. VIBRANT uses a neural network multi-layer perception classifier while VirSorter2 uses a random forest machine learning framework. Besides sequences from NCBI RefSeq, VirSorter2 includes viral sequences of giant viruses mined from public databases, viral sequences from seawater biomes, and novel ssDNA viruses from animals in its training database. Focusing on the differences, VirSorter2 outperformed VIBRANT on seawater and gut biomes while VIBRANT outperformed Virsorter2 on soil biome. The inclusion of uncultivated viral sequences from seawater biomes and human/animal tissues by VirSorter2 might contribute to its better performance on the seawater and gut samples than VIBRANT (Fig. 3A, B, C).

Seeker was the only machine learning tool that did not perform well. Seeker uses much fewer parameters (~ 10^2^) than PPR-Meta and DeepVirFinder (~ 10^6^). The low complexity of the model reflected in the low number of parameters might inhibit the model to capture the nuanced but important patterns in the viral sequences. Besides, like most other tools, Seeker trains on viral and bacterial sequences from NCBI RefSeq, but, differently, Seeker includes many more viral than bacterial genomes in the training databases (2232:75 and 7375:240 viral: bacterial genomes for the first and second training databases, respectively), while the other tools include more bacterial than viral genomes (Additional file 2: Table S1). This class imbalance might lead to loss of important signals and bias the tool.

We further evaluated the true negative rate (TNR, also known as specificity), precision, and F1 score of each tool (Additional file 1: Fig. S3 and Additional file 2: Table S8). The two CNN tools, PPR-Meta and DeepVirFinder, have higher F1 scores than the homology-only tools and other machine learning tools, mostly because of their high sensitivity, but they have lower specificity and lower precision. Homology-only tools tend to be highly specific, but they are not very sensitive and have many false negatives (undetected viral sequences). These properties should be considered when choosing a suitable tool for a specific study. For example, VirSorter2 and VIBRANT were highly specific and relatively sensitive, while PPR-Meta and DeepVirFinder were highly sensitive but less specific than the other tools. The F1 score attempts to capture both these statistics, as the harmonic mean of precision and sensitivity. It is important to note that most machine learning tools were developed more recently than the homology-only tools and therefore included more updated and complete reference databases. All tools can be expected to improve over time as newly discovered viral genomes are added to the training/reference databases. In summary, the classification algorithms, biological signals, and training/reference databases are all crucial factors in determining the performance of the tools. Both PPR-Meta and DeepVirFinder exploited CNNs, indicating the promising potential of CNN algorithms for sequence analysis.

**Fig. 4:**
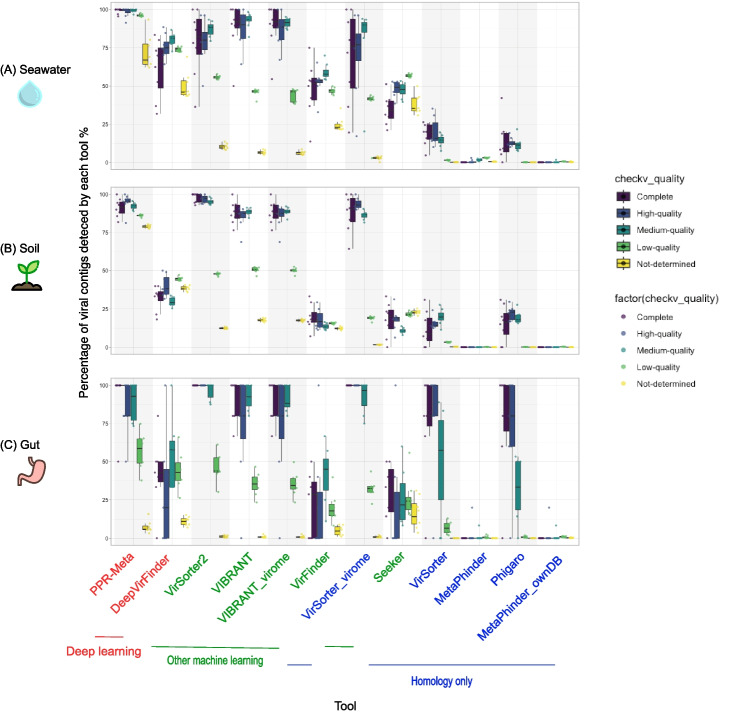
The association of the viral discovery rate with viral contig quality. Percentage of viral contigs detected by each tool from the viral fraction (true positive rate, as known as sensitivity) for viral contigs of different quality from seawater (**A**), soil (**B**), and gut (**C**) biomes, using default cutoffs. The order of the tools on the x-axis and the color of the tool names as in Fig. 3

To investigate influence of the quality of viral contigs on virus discovery, we assessed the quality of the viral contigs using CheckV (Fig. 4). Generally, higher quality viral sequences were easier to detect. CNN and other machine learning tools had higher sensitivity than homology-only tools on all contig quality categories. Compared to homology-only tools, machine learning tools had highly improved virus detecting ability on contigs of medium to complete quality and among which, CNN tools had highly improved virus detecting ability on contigs of low and not determined quality. PPR-Meta had high sensitivity for almost all categories of contig quality. DeepVirFinder had about half the sensitivity of PPR-Meta in all categories. VirSorter2 and VIBRANT had high sensitivity in high-quality viruses (medium to complete), like PPR-Meta, while most low-quality viruses were missed. Detailed information of the CheckV quality results of each contig is deposited into the Zenodo repository (seawater_checkv_quality_summary.tsv, soil_checkv_quality_summary.tsv, and gut_checkv_quality_summary.tsv).

### The effect of adjusting score thresholds

To further investigate the distinguishing ability of each tool, we performed receiver operating characteristics (ROC) analysis. Only tools that provided a virus score were analyzed. We first grouped ROC curves by biome (Fig. 3D, E, F). For the seawater and soil biomes, PPR-Meta had the best virus discovery performance, followed by DeepVirFinder and VirSorter2. For the gut biome, VirSorter2 had the best performance, followed by DeepVirFinder and VirFinder. The results of high performance of PPR-Meta on soil datasets (AUC 0.95, Additional file 2: Table S9) were from both the good distinguishing ability of the tool and the quality of the testing datasets. Technically, the slightly lower performance on seawater datasets (AUC 0.90) could be a result of the tradeoff between sensitivity and specificity of the tool. Biologically, it could be a result of the prevalence of inactive prophages in the microbial fraction, which could have led to the detection of contigs from the microbial fraction which are counted as false positives in our benchmark (see Figs. 2 and 3A, B, C). The homology-only tool MetaPhinder performed no better than chance level based on the ROC curves.

We also grouped ROC curves by tool and colored the curves based on the cutoff score to investigate the optimal cutoff for different tools on different biomes (Additional file 1: Fig. S4). The results show that adjusting virus score thresholds could enhance the virus discovery rate by some of the tools. For example, based on default thresholds, PPR-Meta and DeepVirFinder had high TPR and relatively low FPR when applied to soil datasets. Their thresholds could be decreased with a relatively low risk of false discovery so that more divergent viruses can be found. The choices of tools and thresholds depend on the goals of the study. Researchers who want to detect more novel viruses can use more sensitive tools and decrease the thresholds from the defaults, such as PPR-Meta, DeepVirFinder, and VirFinder. Researchers who have more conservative goals can use more specific tools and increase the thresholds from the defaults, such as VirSorter2.

### Agreement and disagreement between tools

**Fig. 5:**
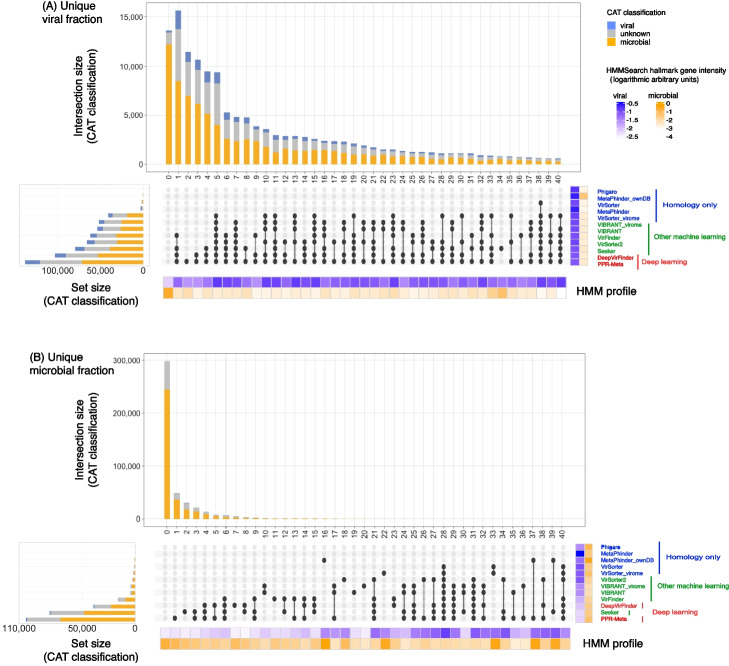
UpSet plots summarizing the overlap in predictions between tools for the viral (**A**) and microbial (**B**) contigs from the seawater samples. The total number of identified viral contigs per tool is shown in the stacked bar plots on the left. Stacked bars above the upset plots visualize the number of viral contigs that were exclusively identified by each tool or tool combination. The left-most stacked bars show the number of contigs that were not identified as viruses by any of the tools. The CAT classification of the contigs is indicated as colors in the bar plots: blue represents contigs classified as viruses, orange represents contigs classified as “Bacteria,” “Archaea,” or “Eukaryota,” and gray represents “no support” or “nan” classifications. Heatmaps below and right of the upset plots visualize the frequency of viral (blue) or microbial (orange) hallmark genes (logarithmic arbitrary units, see the “Methods” section). The intensity of hallmark gene HMM profiles was determined by dividing the length sum of all the HMM hits by the contig length. Color of the tool names as in Fig. 1. For similar plots based on the soil and gut datasets, see Additional file 1: Figs. S5 and S6, respectively

To investigate the agreement and disagreement between tools, we used UpSet plots to show the intersections of identified contigs from the two size fractions of seawater (Fig. 5), soil (Additional file 1: Fig. S5), and gut (Additional file 1: Fig. S6). Tools using similar algorithms clustered together and had more linkages in the UpSet matrices, indicating that the annotations of these tools tend to agree with each other. CNN tools identified the most viral contigs from the viral fractions and agreed with each other as they clustered on the bottom of the UpSet matrices. As expected, most microbial contigs were not identified as viral by any tool (the left-most stacked bars are the tallest among all bars in the B panels). More consistency of predictions (more linkages in the UpSet matrices) was seen in the viral fractions (A panels) than in the microbial fractions (B panels), indicating that tools tend to agree with each other more on viral than on microbial contigs. For seawater and soil biomes, most viral contigs were identified by at least one tool, but this was not the case for the gut biome (the left-most stacked bar is the tallest among all bars in the A panel in Fig. S6), again suggesting that possible microbial contamination in the gut viral datasets introduced negative (microbial) sequences into the positives (viral) contig set.

### Validation of predicted viral contigs in the real-world testing dataset

To assess the purity of the microbial and viral fractions from each biome, we further characterized the contigs using taxonomic and functional annotation tools with CAT and hmmsearch, respectively (Fig. 5 (seawater), Additional file 1: Fig. S5 (soil), and S6 (gut)). CAT is a tool to predict open reading frames from assembled contigs and search their homology to the NCBI non-redundant protein database to assign taxonomy to contigs. Hmmsearch searches viral and microbial profile HMMs from assembled contigs. Both tools gave an overall trend of viral and bacterial protein signals found from the contigs. Generally, more viral signals were found in the viral fraction than in the microbial fraction; more microbial signals were found in the microbial fraction than in the viral fraction. The stacked bars above and left of the UpSet matrices visualized the CAT classification on the contig subsets. More contigs in viral fraction than in microbial fraction were classified as viral by CAT (more blue color bar in the viral fraction), confirming an enrichment of viral sequences in the viral fraction. The heatmaps below and right of the upset plots show to what extent the contig subsets contained viral or microbial marker gene HMM profiles. Viral markers were found in both the viral and microbial fractions but more in viral fraction (the intensity of blue color is stronger in the viral fraction than in the microbial fraction), again confirming an enrichment of viral sequences in the viral fraction. Microbial markers were mostly found in the microbial fraction (the intensity of orange color is stronger in the microbial fraction than in the viral fraction), except for gut biome that contained a lot of microbial markers in the viral fraction (Additional file 1: Fig. S6).

Viral contigs that were identified by many tools showed great CAT viral protein signals and/or viral marker gene HMM profile signals, such as column 5 and 15 in Fig. 5A. The coincide of strong viral signals and contigs identified by many tools indicated that tools agreed with each other more when the conventional viral signals are strong. A strong viral signal was also found in some microbial contigs, such as column 28 in Fig. 5B which contains contigs predicted as viral by nine different methods. This viral signal from microbial fraction contigs could indicate prophages, free virions, or other viral elements that could not be removed by prior experimental and bioinformatic processes. A summary of viral and microbial signals on contig subsets can be found in Additional file 2: Table S10 and S11. Detailed CAT classification and hmmsearch results can be found in the Zenodo repository (seawater_cat_taxonomy_official.txt, soil_cat_taxonomy_official.txt, gut_cat_taxonomy_official.txt, seawater_hmmsearch_domtblout.txt, soil_hmmsearch_domtblout.txt, and gut_hmmsearch_domtblout.txt).

**Fig. 6:**
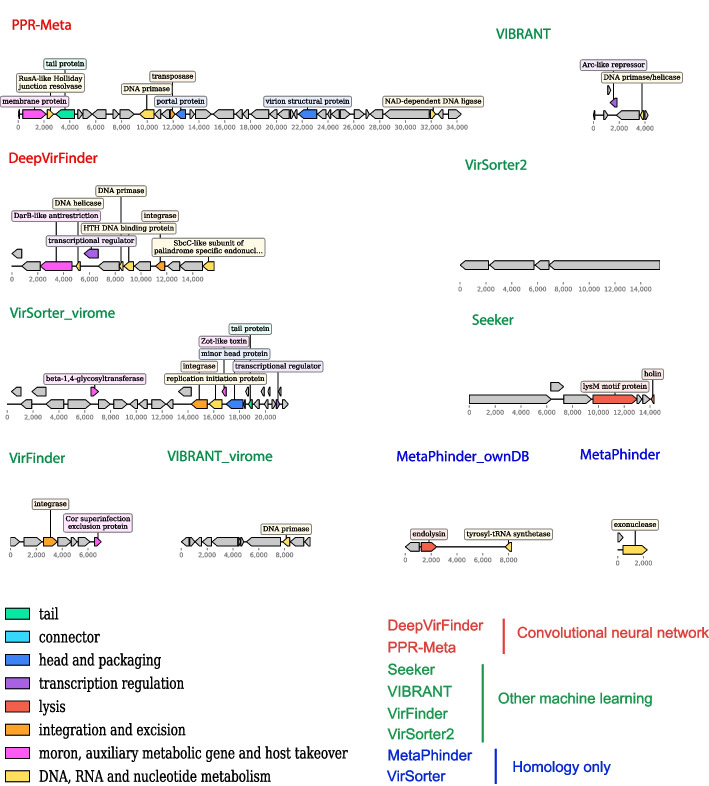
Genomic maps of the longest contigs that were exclusively identified by individual tools in the seawater virome dataset. For similar plots based on the soil and gut datasets, see Additional file 1: Figs. S7 and S8, respectively

To further investigate whether the identified contigs represent real viral sequences, the longest viral contigs that were exclusively identified by each tool in each biome were extracted, classified, and annotated. PhaGCN2 placed 6/31 of these contigs into the viral taxonomy (Additional file 2: Table S12). Although the remaining 25 contigs were not taxonomically classified, they did contain viral hallmark genes, such as genes encoding for portal proteins, head scaffolding proteins, and tail related proteins (Fig. 6 (seawater), Additional file 1: Figs. S7 (soil) and S8 (gut)), suggesting that these contigs are derived from real novel viruses. This analysis indicates that all tools have their strengths and weakness. Some tools may not be able to predict a wide range of viruses, but almost all tools, except for Sourmash, still identified certain viral sequences that the other tools missed.

### Benchmarking tools on the simulated testing datasets derived from the RefSeq database

We constructed a simulated dataset with the viral (6,155 sequences) and bacterial genomes (22,552 sequences) deposited into the RefSeq database after the last tool (VirSorter2) training database was created. We calculated similarity scores of these viruses (new viruses) based on their similarity compared to the viruses (old viruses) deposited before the last tool training database was created (Additional file 2: Table S13). We cut simulated contigs from the viral and bacterial genome sequences and input them for virus identification using the nine tools. As above, CNN tools outperformed homology-only tools (Additional file 1: Fig. S9A, B and Additional file 2: Table S14). This strong performance did not depend on the degree of similarity of the new virus genome to the older ones (Additional file 1: Fig. S9C and Additional file 2: Table S15). CNN tools even outperformed homology-only tools on the viruses with relatively high similarity with known viruses. This could be because the CNN tools were built after homology-only tools and thus contained more and more diverse reference viruses in their training datasets. PPR-Meta performed the best among all tools on the simulated data, but with a lower discovery rate than in the real-world metagenomic data (Fig. 3A, B, C). This could be due to the short sequence lengths of the mock contigs (mean: 2000 bp) compared to the real-world contigs (Additional file 1: Fig. S2). Seeker performed better on the simulated contigs than on the real-world contigs. Detailed results per contig are available in the Zenodo repository (simulated_bacterial_fragments_wtp_output.txt, simulated_viral_fragments_wtp_output.txt).

### Computational resources used for each tool

This study was performed on a shared high performance computing cluster. We benchmarked the computational resources used for each tool on the smallest dataset from the seawater biome (188,159 contigs, 164 MB) in a high-performance cluster of 48 Gold-6240R CPUs and 503.5 GB RAM or 48 Gold-6240R CPUs and 1510.5 GB RAM. We captured the CPU usage, physical memory usage and execution time in Additional file 2: Table S16. DeepVirFinder and VirFinder had the largest and smallest computational footprints, respectively. Two tools with the best performance, PPR-Meta and VirSorter2, used intermediate computational resources. PPR-Meta is fast but relatively memory- and CPU-intensive, while VirSorter2 is relatively slow but requires less memory.

## Discussion

### Prior benchmarking studies

Several studies have compared the performance of bioinformatic virus identification tools. All studies benchmarked different sets of tools with some of them having overlaps on tested tools. One of them evaluated tools only by describing the methods of the workflows without quantification analysis [48]. Another one benchmarked tools that specialize in identifying viruses from clinical samples [51]. Four benchmarking works [49, 50, 52, 53] mainly used simulated viral and non-viral testing datasets that were sampled from publicly available complete viral and microbial genomes (e.g., NCBI RefSeq). A summary of the tested tools and testing datasets of each study can be found in Additional file 2: Table S2.

All these studies gave insights about tool performance and which tools to choose for virus identification. Ho et al. 2021 found that machine learning-based tools outperformed homology-only tools on the simulated dataset, which was consistent with our benchmarking results [53]. Besides a simulated dataset, they benchmarked tools on a mock community dataset consisting of five phage strains. Most tools performed significantly worse on the mock community dataset than on the simulated dataset derived from reference genomes, illustrating how simulated data sampled from RefSeq could overestimate tools’ performance. Pratama et al. 2021 found that viral identification efficiency increases with fragment length, and almost all tools can correctly identify viral contigs of 10 kb or longer [50]. “Gene content based” tools (VirSorter) can maximize the true positive rate and minimize the false positive rate at length > 3 kb. K-mer-based tools (DeepVirFinder) and BLAST-based tools (MetaPhinder) were good at identifying viruses from short (< 3 kb) viral genome fragments. Complementary to their results, we found that the viral identification sensitivity increases with the quality of contigs (Fig. 4). High-quality contigs were better identified by machine learning tools than by homology-only tools. CNN tools were better at identifying low-quality contigs than other machine learning tools. The most recent benchmarking work by Schackart et al. 2023 used simulated datasets as well as a real-world gut metagenome dataset [52]. Their main findings agreed with us—that homology tools (VirSorter, VIBRANT, and VirSorter2) demonstrate low false positive rates and robustness to eukaryotic contamination, while machine learning tools (VirFinder and DeepVirFinder) have high sensitivity, allowing them to identify phages that are distinct from those in the reference databases.

For benchmarking studies that simulated testing datasets, while an effort was made to benchmark the tools on data that was not part of the reference/training dataset, there still might be instances of overlap. For example, Glickman et al. 2021 used VirSorter to perform prophage identification during testing dataset preparation, which might provide VirSorter with an advantage over other tools in prophage identification in this benchmarking work [49]. And indeed, VirSorter performed the best on identifying phages based on F1 score in this study.

### The advantages and limitations of using real-world metagenomic data

The usage of newly generated metagenomic data as testing datasets in our benchmark, including a marine Antarctic dataset from an understudied region, allowed us to avoid overlap between testing and training/reference datasets. Besides, the usage of real-world metagenomic testing datasets gives our study the perspective of metagenomic virus discovery. The main limitation of our benchmarking study is that we are not completely certain about the purity of our ground truth viral (< 0.22 μm) and microbial (> 0.22 μm) datasets.

To address this, we carefully selected metagenomic data from the studies with appropriate standard operation procedures to separate viruses from microbes. Both our dataset [64] and the publicly available datasets [65, 66] used in this benchmark performed a DNase treatment in the viral fraction before DNA extraction from virions, minimizing free microbial DNA contamination [62, 67]. The DNase treatment effectively removes free microbial DNA in the viral fraction and is commonly used in virome studies [68–73]. We also assessed the contamination of microbial DNA in the viral size fraction by using ViromeQC (Additional file 2: Table S5). The microbial size fraction could contain viral sequences, including integrated prophages or viruses that are attached to cells or large debris or particles. This was illustrated by the viral signals found by CAT and hmmsearch in the microbial datasets (Fig. 5, Additional file 1: Fig. S5 and S6). However, more viral signals were found in the viral fraction than in the microbial fraction, confirming the enrichment of viruses in the viral fraction and the depletion of viruses in the microbial fraction. We recognize that integrated prophages that are not active as free virions might still be identified as viruses in the microbial fraction (spurious false positives).

Besides viruses, mobile genetic elements (MGEs) such as plasmids, insertion sequence elements, integrative and conjugative elements, and integrons are also prevalent in the environment, but it can be difficult to distinguish these MGEs, even when using specialized bioinformatic tools [74]. This does not necessarily mean that the tools perform poorly but also suggests that recombination between MGEs is rampant. While our benchmarking work did not target other MGEs such as plasmids or prophages specifically, we minimized their influence by identifying and removing any homologous contigs shared between the viral and microbial fractions (Fig. 1). We attempted to taxonomically classify some of the identified viral contigs using PhaGCN2 [75], but most of them remained unclassified.

While other benchmarking studies that used simulated data had more purified data, this was at the expense of vastly reduced viral/microbial community complexity, since sequences were only sampled from a fixed set of genomes. Strain-level microdiversity, which is an important parameter in viral ecology [76], is not represented in such simulated data. In summary, our benchmarking is complementary to the benchmarking work done by others. The usage of real-world metagenomic data from different biomes ensured the high complexity and reality of the testing datasets and avoided the problems of overlap between training and testing datasets, albeit with the compromise of not knowing the exact compositions of the testing datasets.

We benchmarked tools on three distinct biomes, seawater, soil, and human gut. We chose these three biomes because they differed greatly on microbial community compositions and diversity and represent very different microbial compositions when viewed in the context of global microbiome datasets [56]. While arguably, samples from three individual studies cannot be assumed to represent all microbiomes, the different tools have similar performance in the different biomes, indicating that our results are generalizable.

## Conclusions

This study benchmarks the performance of nine state-of-the-art bioinformatic virus identification tools using real-world metagenomic data. To evaluate how the tools perform on datasets from different biomes, we used datasets from three distinct biomes, i.e., seawater, soil, and gut. As CNN tools outperformed homology-only tools, especially on contigs of low quality, this seems to be a very promising avenue for virus mining, and more advances are expected as this field matures. PPR-Meta, DeepVirFinder, VirSorter2, and VIBRANT performed the best among all benchmarked tools with relatively high true positive rates and relatively low false positive rates. No tool is perfect; every tool has its own strengths and weaknesses. However, it is not recommended to use union of all tools as some of the tools identified many false positives. The selection of a tool/tools may depend upon the desired application as well. PPR-Meta and DeepVirFinder were found to be sensitive but not as precise as some other tools. Thus, they can be used when detection of novel viruses is important and false positives are perhaps less of an issue. VirSorter2 and VIBRANT were more precise (fewer false positives) but also less sensitive compared to PPR-Meta and DeepVirFinder. Thus, they can be applied to studies when precision is more important than sensitivity. The adjustment of the cutoff virus scores also plays a role in the distinguishing ability of tools. If possible, in the experimental setup, we suggest using a small dataset of pairs of viral and microbial datasets for at least some samples to explore the optimal thresholds of virus detection tools using a setup as we followed in our study. For this, we have made our full Snakemake pipeline [77, 78] available. Besides, the experimental flow of obtaining viromes and the quality of assembled contigs would influence the discovery performance of virus identification tools.

Our comprehensive analysis of viral identification tools to assess their performance in a variety of biomes provides valuable insights to viromics researchers looking to mine viral elements from novel metagenomic data across biomes. We hope that the results of this benchmarking work will provide researchers with a guide to selecting the appropriate tool and adjusting parameters for their own viral identification research.

## Methods

### Construction of testing datasets from real-world metagenomic data across biomes

To investigate how the tools perform on datasets from different biomes, we selected datasets from Antarctic seawater (this study) [54], tomato soil [31], and human gut [55] (Additional file 2: Table S3). We collected a total of 48 metagenome datasets, including eight paired viral and microbial datasets from each biome (Additional file 2: Table S4). The viral datasets were obtained by size fractionation through a 0.22 μm membrane followed by DNase treatment to remove free DNA, reducing the likelihood of contaminating the viruses with sequences derived from cellular organisms. To further reduce biases in the detected viruses, we selected datasets where the DNA was not amplified prior to library preparation and sequencing [79, 80]. In all studies, total DNA for the microbial fraction was extracted with the PowerSoil kit. Paired-end sequencing was performed on the Illumina platform [31, 54, 55]. As these microbial samples might still contain some viral sequences and the viral samples might contain some microbial sequences, any overlapping sequences between viral and microbial datasets will be removed below. The quality of each viral dataset was assessed by ViromeQC (v1.0.1) [81], which quantifies viral enrichment in viral samples by calculating three enrichment scores based on reads mapping to the small and large subunit rRNA, and single-copy bacterial markers. Raw reads were assessed and quality-controlled using fastp (v0.22.0) [55, 82] and MultiQC (v1.11) [83] and assembled using metaSPAdes (v3.15.3) [84] with k-mer sizes of 21, 33, 55, 77, 99, and 127. Contigs < 1500 bp were removed before downstream analysis using seqtk (v1.3) [85]. Raw sequencing reads were mapped back to assembled contigs with lengths of at least 1500 bp using bwa mem (v0.7.17) [86], and the mapping statistics were summarized using samtools stats (v1.14) [87].

Overlapping sequences between viral and microbial fractions were identified by mapping the viral to the microbial contigs using Minimap2 (v2.22) [88] with arguments -x ava-ont for contigs. Minimap2 does not have a specific identity cutoff but is designed for mapping long reads with 5–15% mismatches. Contigs with Minimap2 hits covering at least 80% of the microbial contig length were removed from either size fraction.

We used unique contigs with lengths of at least 1500 bp from the viral and microbial size fractions as ground truth positives and negatives, respectively. Viral contigs that were identified as viral and non-viral by the tools were regarded as true positives and false negatives, respectively. Microbial contigs that were identified as viral and non-viral were regarded as false positives and true negatives, respectively (Fig. 1).

### Construction of the simulated testing datasets from the RefSeq database

To investigate how the tools perform on mock data with known composition, we downloaded 6495 viral and 52,046 bacterial reference genomes deposited in the RefSeq database after 12 January 2020, when the last tool training database, VirSorter2, downloaded their training dataset (latest date: 13 November 2023). We removed plasmids and genomes shorter than 1500 bp, resulting in 6155 viral and 22,552 bacterial genomes. To assess the similarity of the viruses deposited after 12 January 2020 to the ones deposited before that date, we performed an mmseqs2 easy search with the translated type (–search-type 2). Based on this search, we grouped the viruses into three categories, with low (< = 20% similarity, *n* = 41,970), medium (> 20% and < = 40% similarity, *n* = 15,580), and high (> 40% and < = 100% similarity, *n* = 4,000) identity to older RefSeq viruses. We cut 52,406 fragments (mock contigs) with lengths according to a normal distribution with mean 2000 bp, standard deviation 500 bp, and minimum 1500 bp from both the viral and bacterial datasets.

### Benchmarked tools

We predicted viral contigs using the What-the-Phage pipeline (v1.0.2) [89], which is a wrapper of ten virus identification tools. VirNet was not run successfully. The nine remaining tools included CNN tools—DeepVirFinder v1.0 [41] and PPR-Meta v1.1 [42], other machine learning tools—Seeker with no release version [90], VIBRANT v1.2.1 (with and without virome mode) [43], VirFinder v1.1 [40], andVirSorter2 v2.0 [44], and homology-only tools—MetaPhinder [38] with no release version (with and without own database), Sourmash v2.0.1 [39], and VirSorter v1.0.6 (with and without virome mode) [37] (Additional file 2: Table S1). The predicted classes were binarized with 0 and 1 corresponding to predicted microbial and viral origin, respectively. Our benchmarking work used contigs with lengths of at least 1500 bp because this is the minimum length of the input sequence that can be handled by all tools. Some tools had additional requirements. For example, VIBRANT requires input sequences of at least four open reading frames. Thus, not all contigs were given a prediction result. Contigs that were not given any prediction were assigned to NAs.

### Tool performance analysis

NAs of the identification results by each tool were replaced by zero (i.e., not predicted as viral) before calculating performance measures. Performance measures, including true positive rate (TPR, also known as sensitivity, Eq. 1), false positive rate (FPR, Eq. 2), true negative rate (TNR, also known as specificity, Eq. 3), precision (Eq. 4), and F1 score (Eq. 5) were calculated and plotted in box plots. Receiver operation curves and UpSet plots were created using the summarized statistics.

1 $$\mathrm{TPR\ or\ sensitivity}=\frac{{\text{TP}}}{{\text{TP}}+{\text{FN}}}$$

2 $${\text{FPR}}=\frac{{\text{FP}}}{{\text{FP}}+{\text{TN}}}$$

3 $$\mathrm{TNR\ or\ specificity}=\frac{{\text{TN}}}{{\text{TN}}+{\text{FP}}}$$

4 $${\text{precision}}=\frac{{\text{TP}}}{{\text{TP}}+{\text{FP}}}$$

5 $${\text{F}}1\mathrm{\ score }=\frac{2*{\text{precision}}*{\text{sensitivity}}}{{\text{precision}}+{\text{sensitivity}}}$$

In addition, we assessed to what extent the contigs from the viral fraction represented (in-)complete genomes using CheckV (v1.0.1) [91] and plotted the detected viral ratio in each quality rank in box plots.

### Additional contigs validation

We taxonomically classified the contigs in all testing datasets using CAT (v5.2.3) [92] with Diamond (v2.0.9) [93] and the CAT_database.2021–04-30 database. CAT add_names with the –only_official argument and CAT summarize were used to process the output. Only classifications at the superkingdom rank were used, including “Bacteria,” “Archaea,” “Eukaryota,” “Viruses,” “no support,” and “NA.” “No support” in CAT means that the ORFs in the contigs had no hit in the database. Since viruses are relatively unexplored, these contigs might be derived from novel viruses [29]. “NA” means that the ORFs had hits in the database, but these have conflicting taxonomic annotations, for example if different ORFs on a given contig map to viruses and bacteria, potentially reflecting prophages on microbial contigs. We interpreted the contigs with CAT classifications “Viruses” as potentially viral, “Bacteria,” “Archaea,” and “Eukaryota,” as microbial, and “no support” and “NA” as unknown. The distributions of viral and microbial contigs identified by CAT in each dataset collection of contigs exclusively identified by tools or tool combinations were shown by bar plots.

To further validate the contigs in the testing datasets, we queried them for viral and microbial signals using 8773 viral and 7185 microbial HMMs from CheckV (v1.4) [91]. We translated the nucleotide contigs into amino acid sequences in six frames using transeq from EMBOSS (v6.6.0) [94] and searched the sequences for the 15,958 marker HMMs using hmmsearch (v3.2.1) [46, 95]. To quantify the overall viral/microbial signal in each subset of contigs, we calculated the fraction of the contig lengths that was covered by the HMM hits, converted the fraction to log ratios, and visualized the results in heatmap.

To further investigate specific sequences that were exclusively predicted by different tools, we taxonomically classified them using PhaGCN2.0 (v2.0) [75]. Gene annotation plots were created by a custom python script, after searching the translated amino acid sequences against the PHROG (v4) [96] HMM profile database using hhsearch from the HH-suite (v.3.3.0) [97].

All statistics were calculated and plotted using custom python and R (v4.1.0) scripts and using R packages dplyr (v1.0.8), tidyverse (v1.3.1), scales (v0.6.0), ggplot2 (v3.3.5), ggbeeswarm (v1.1.1), ROCR (v1.0.11) [98], UpSetR (v1.4.0) [99], ComplexHeatmap (v2.8.0) [100], and ggpattern (v0.4.2).

### Supplementary Information

**Additional file 1: Fig. S1**. Snakemake workflow of the pipeline. **Fig. S2**. Real-world metagenomic assembled contigs number and length distribution. **Fig. S3**. True negative rate, precision, and f1 score of tools. **Fig. S4**. Receiver operating characteristiccurves per tool. **Fig. S5**. UpSet plots summarizing the overlap in predictions between tools from the soil samples. **Fig. S6**. UpSet plots summarizing the overlap in predictions between tools from the gut samples. **Fig. S7**. Genomic maps of the exclusively identified, longest contigs in the soil virome dataset. **Fig. S8**. Genomic maps of the exclusively identified, longest contigs in the gut virome dataset. **Fig. S9**. Performance of tools on simulated data.

**Additional file 2: Table S1**. Summary of virus identification tools. **Table S2**. Summary of existing benchmarking work. **Table S3**. Summary of generation methodologies of selected real-world metagenomic datasets. **Table S4**. Sample metadata and data availability of selected real-world metagenomic datasets. **Table S5**. ViromeQC results of selected real-world metagenomic datasets. **Table S6**. Summary of real-world metagenomic assembled contigs numbers. **Table S7**. Reads mapping summary. **Table S8**. Confusion matrix of real-world metagenomic testing datasets. **Table S9**. AUC of tools per biome. **Table S10**. CAT classification summary per biome. **Table S11**. HMM coverage per biome. **Table S12**. PhaGCN2 results of the exclusively identified, longest contigs. **Table S13**. Virus similarity scores based on their similarity compared to previously deposited viral genomes. **Table S14**. Confusion matrix of simulated testing datasets. **Table S15**. Number of detected viruses from each similarity category by tools. **Table S16**. Computational resources used per tool.

**Additional file 3. **Review history.

## Data Availability

The raw sequences from Antarctic seawater are available at National Center for Biotechnology Information under accession number PRJEB71789 (https://www.ebi.ac.uk/ena/browser/view/PRJEB71789) [64]. The raw sequences from the soil biome are available at National Center for Biotechnology Information under accession number PRJNA646773 (https://www.ncbi.nlm.nih.gov/bioproject/PRJNA646773) [65]. The raw sequences from the gut biome are available at National Center for Biotechnology Information under accession number PRJNA389927 (https://www.ncbi.nlm.nih.gov/bioproject/PRJNA389927) [66]. More information about the SRA accession numbers is available in Additional file 2: Table S4. Assembled contigs from the eight pairs of viral and microbial samples of the three biomes, simulated contigs derived from the RefSeq database, and other supporting files can be found in the Zenodo repository: 10.5281/zenodo.10886947. Our full pipeline is integrated into a Snakemake (v6.5.1) [101] workflow [77, 78] (Additional file 1: Fig. S1). All scripts can be found in GitHub repository https://github.com/MGXlab/virus_identification_tools_benchmarking.
