# Supplementary material for: Benchmarking bioinformatic virus identification tools using real-world metagenomic data across biomes
Source: Genome Biol. 2024 Apr 15;25:97. doi: 10.1186/s13059-024-03236-4 (PMC11020464; doi:10.1186/s13059-024-03236-4)
Supplement: Supplementary file 1 — Additional file 1: Fig. S1. Snakemake workflow of the pipeline. Fig. S2. Real-world metagenomic assembled contigs number and length distribution. Fig. S3. True negative rate, precision, and f1 score of tools. Fig. S4. Receiver operating characteristiccurves per tool. Fig. S5. UpSet plots summarizing the overlap in predictions between tools from the soil samples. Fig. S6. UpSet plots summarizing the overlap in predictions between tools from the gut samples. Fig. S7. Genomic maps of the exclusively identified, longest contigs in the soil virome dataset. Fig. S8. Genomic maps of the exclusively identified, longest contigs in the gut virome dataset. Fig. S9. Performance of tools on simulated data. [file 13059_2024_3236_MOESM1_ESM.docx]

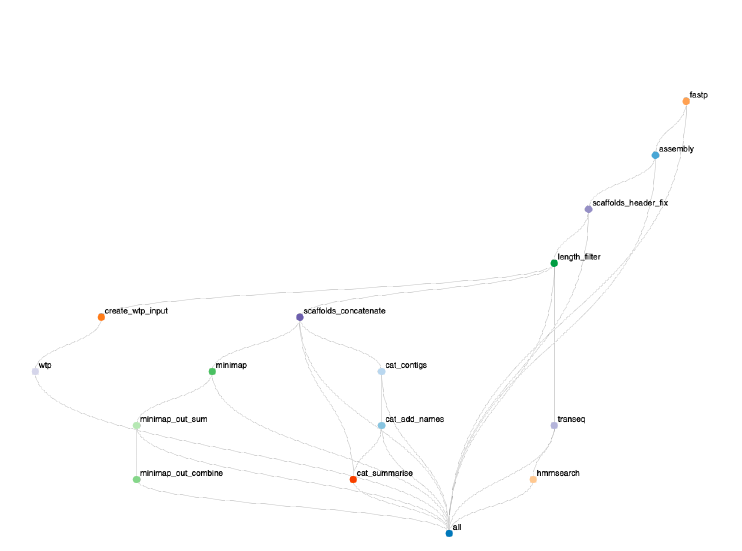


**Fig. S1** Snakemake workflow of the pipeline to assess the quality of the raw reads, assemble the quality-control filtered reads into contigs, filter contigs based on lengths, cluster contigs from two size fractions to remove homologous contigs, run bioinformatic virus identification tools (CAT and hmmsearch) in parallel, and further validate the contigs using extra bioinformatics tools.


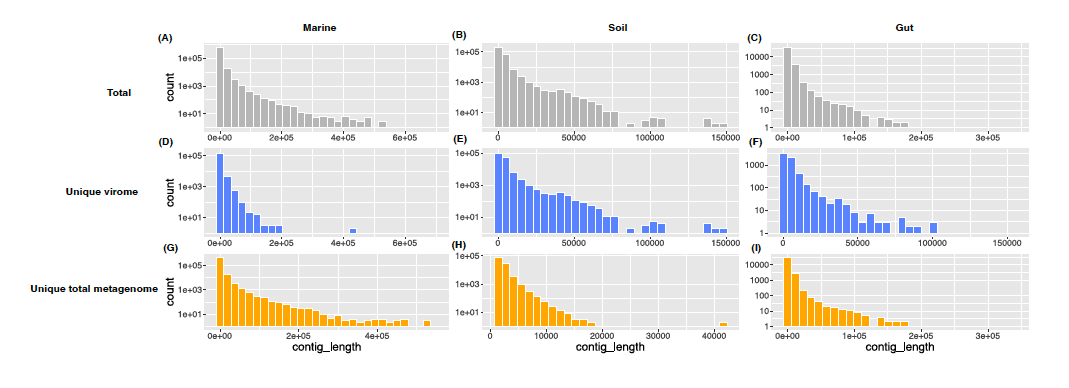


**Fig. S2** Length distributions of all (A, B, C), unique viral (D, E, F), and unique microbial (G, H, I) contigs from seawater (A, D, G), soil (B, E, H), and gut (C, F, I) samples. Y axes are in log scales.


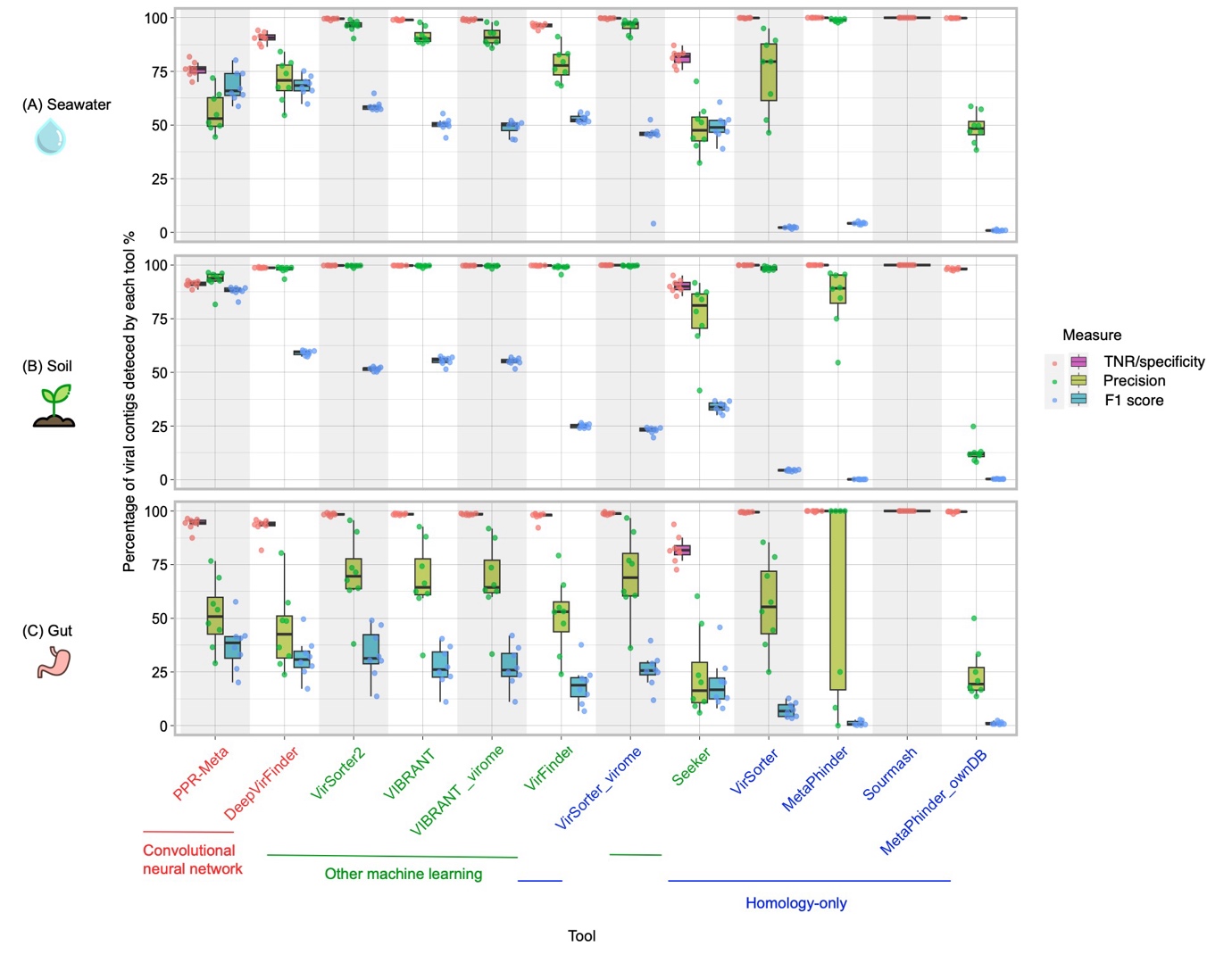


**Fig. S3** True negative rate (TNR, also known as specificity), precision, and F1 score of each tool on samples across seawater (A), soil (B), and gut (C) biomes based on tools’ default cutoffs. Sourmash did not have a precision or F1 score because it did not detect any virus.

The order of the tools on the x-axis and the color of the tool names as in Fig. 3.

**Fig. S4** Receiver operating characteristic (ROC) curves per tool of (A) PPR-Meta, (B) DeepVirFinder, (C) VirFinder, (D) VirSorter2, (E) Seeker, (F) MetaPhinder, and (G) MetaPhinder with own database. The curves from outside to inside are for soil, seawater, and gut biome, respectively. Asterisks are the default cutoffs (values shown in the header of each panel) of tools. The color scheme showed all the possible cutoffs of each tool. The area under the ROC curve (AUC) of each tool is listed in Additional file 2: Table S9.


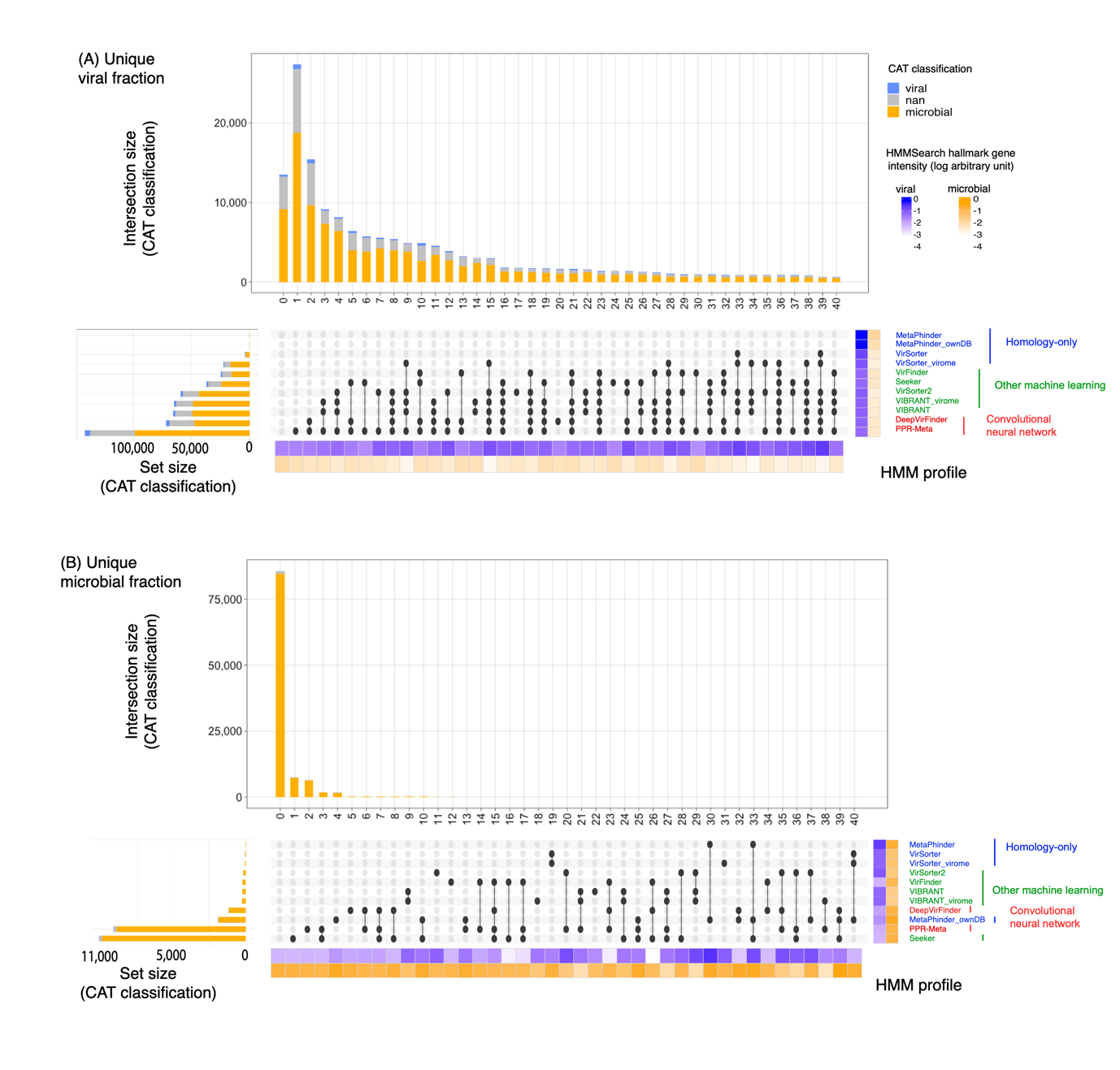


**Fig. S5** UpSet plots summarizing the overlap in predictions between tools for the viral (A) and microbial (B) contigs from the soil samples. The total number of identified viral contigs per tool is shown in the stacked bar plots on the left. Stacked bars above the upset plots visualize the number of viral contigs that were exclusively identified by each tool or tool combination. The left-most stacked bar shows the number of contigs that were not identified as viruses by any of the tools. The CAT classification of the contigs is indicated as colors in the bar plots: blue represents the contigs classified as viruses, orange represents contigs classified as “Bacteria”, “Archaea”, or “Eukaryota”, gray represents “no support” or “nan” classifications. Heatmaps below and right of the upset plots visualize the frequency of viral (blue) or microbial (orange) hallmark genes (logarithmic arbitrary units, see Methods). The intensity of hallmark gene HMM profiles was determined by dividing the length sum of all the HMM hits by the contig length. Color of the tool names as in Fig. 1.


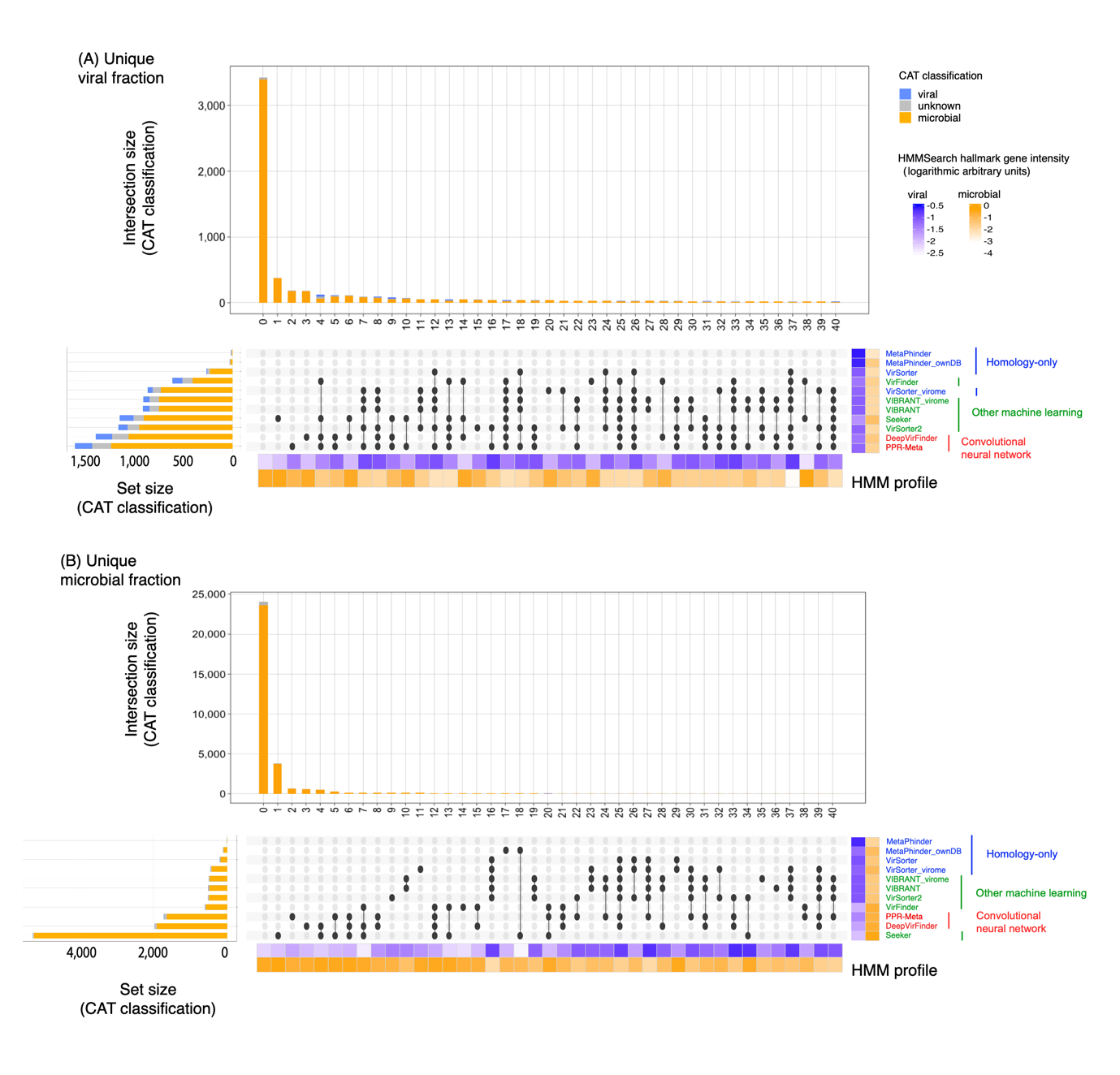


**Fig. S6** UpSet plots summarizing the overlap in predictions between tools for the viral (A) and microbial (B) contigs from the gut samples. The total number of identified viral contigs per tool is shown in the stacked bar plots on the left. Stacked bars above the upset plots visualize the number of viral contigs that were exclusively identified by each tool or tool combination. The left-most stacked bar shows the number of contigs that were not identified as viruses by any of the tools. The CAT classification of the contigs is indicated as colors in the bar plots: blue represents contigs classified as viruses, orange represents contigs classified as “Bacteria”, “Archaea”, or “Eukaryota”, gray represents “no support” or “nan” classifications. Heatmaps below and right of the upset plots visualize the frequency of viral (blue) or microbial (orange) hallmark genes (logarithmic arbitrary units, see Methods). The intensity of hallmark gene HMM profiles was determined by dividing the length sum of all the HMM hits by the contig length. Color of the tool names as in Fig. 1.

**Fig. S7** Genomic maps of the longest contigs that were exclusively identified by individual tools in the soil virome dataset.

**Fig. S8** Genomic maps of the longest contigs that were exclusively identified by individual tools in the gut virome dataset.

**Fig. S9** Performance of tools on simulated data. (A) Percentage of contigs identified in the viral (true positive rate, blue) and bacterial (false positive rate, orange) datasets. (B) Specificity, precision, and F1 score of each tool. (C) Number of viruses detected by each tool from viral sequences with low, medium, high, and unknown similarity with previously deposited viral sequences in the RefSeq database. The order of the tools on the x-axis and the color of the tool names as in Fig. 3.
