# Supplementary material for: Benchmarking bioinformatic virus identification tools using real-world metagenomic data across biomes
Source: Genome Biol. 2024 Apr 15;25:97. doi: 10.1186/s13059-024-03236-4 (PMC11020464; doi:10.1186/s13059-024-03236-4)
Supplement: Supplementary file 3 — Additional file 3. Review history. [file 13059_2024_3236_MOESM3_ESM.docx]

Review History

**First round of review**

**Reviewer 1**

**Are you able to assess all statistics in the manuscript, including the appropriateness of statistical tests used?** Yes, and I have assessed the statistics in my report.

**Comments to author:**

Please consider the following questions:

- Are the methods appropriate to the aims of the study, are they well described, and are necessary controls included? If not, please specify what is required.

needs more work. see below for details.

- Are the conclusions adequately supported by the data shown? If not, please explain

needs more work. see below for details.

- Are sufficient details provided to allow replication and comparison with related analyses that may have been performed? If not, please specify what is required.

Yes.

- Does the work represent a significant advance over previously published studies?

yes, if concerns can be addressed.

- Is the paper of broad interest to others in the field, or of outstanding interest to a broad audience of biologists?

yes.

The article by Wu et al. describes the comparison of ten different viral sequence detection pieces of software (tools) using real-life microbial metagenome and virome datasets from three key environments (soil, seawater and human gut). The study reports that the machine learning based tools typically perform better compared to the homology-based tools especially in terms of their sensitivity. That is, the former tools identify more viral sequences that the latter tools do not report as viral for these datasets. I have two major concerns with the results of this study. First, the datasets used for the evaluation are environmental samples, and thus of unknown composition. Accordingly, I don't think it is easy (not possile at all?) to say which tool performed better. A mock sample (of known composition) would be more useful for comparison purposes, I believe. The authors argue against the latter using a couple examples from the literature, but all these examples mentioned were small diversity mock samples, and thus limited, as the authors also suggest. A more appropriate dataset may be to sample 100 to 1000 viral genomes from the public databases, simulate a metagenome from them and then query the tool against these mock datasets while first removing the chosen genomes from the training datasets of the tools and/or the public database the tools are using (and possibly mixing them also with similar microbial datasets to increase complexity/challenge for the tools). Further, one can choose the genomes to represent different levels of novelty compared to the remaining genomes in the database/training dataset (same species; different species of the same family; different families etc.) in order to assess how the tools do with increasing levels of novelty of the target viral sequences.

Second, the comparison found that the machine-learning tools find more viral contigs. However, I believe the only data used to say that these contigs were indeed viral is that the sequences were found in the viral fraction (viromes). However, it is not certain that these sequences are indeed viral; in my view, they could represent anything such as virions, gene transfer agents, other mobile elements, plasmids, junk of viral preps, right? If there is no homology to any available isolate genome, how the authors could be certain that they are indeed viral (and specifically for the purpose of the study bacteriophages), and thus conclude that machine learning tools perform better? I would argue that plasmid and other mobile element sequences often look like phage genomes e.g., have repeats, integrases, lots of hypothetical genes. So, I would not be surprised if the machine learning tools identify as viral such (non-viral) sequences.

Specific/minor comments:

Line 58. Viral genome integration too (prophages).

Line 124. No threshold on identity, e.g. >95% nt identity, or Minimap2 has a default sequence identity threshold?

Methods section: The bioinformatics part looks robust and clear to me overall. But I am not sure about confirming/validating that whether or not a contig is indeed viral, the CheckV is the preferred approach instead, or in addition to, searching against the viral isolate genome database (~3 million genomes available).

Lines 225-228. The major result of the study seems to be that CNN methods find more viral contigs. The only data in support of this appears to be that the sequences were found in the viral fraction but such sequences could be anything, right? Meaning if there is not enough homology to a genome, they could be virions, gene transfer agents, other mobile elements, plasmids, junk of viral preps, correct? Consistent with this assumption, the sequences CNN identified as viral that the homology did not find were marked as NA/not-identified by the later right, not microbial or other origin?

Line 260 and elsewhere. I think it would be useful for the authors to mention, in the main text, what caused the lower specificity of the machine learning tools. Is it that they identified microbial sequences as viral for example? How much/often?

Line 329-331. Have you searched these contigs for matches against plasmids or other mobile elements in the NCBI/UNIPROT databases? And, plasmid is just one of the possibilities if the query sequences are non-viral, not all possibilities.

Line 346. I would not call RefSeq sequence data "artificial" (they are real data). Maybe "test dataset of selected reference genome/sequences"?

**Reviewer 2**

**Are you able to assess all statistics in the manuscript, including the appropriateness of statistical tests used?** No, I do not feel adequately qualified to assess the statistics.

**Comments to author:**

Thank you for giving the nice overview of the state-of-the-art viral prediction tools and their performance on real-world metagenomic data.

My main concern is the use of datasets from only three biomes - it calls into question the possible generalization of your findings.

Another potential issue is the potenital non-viral contigs in size-fractionated samles (virions, plasmids and other entities from the dark corners of virosphere), as well as possible viral fraction in bacterial samples

Also, in a benchmarks like that I always curious about this binary opposition "viral/bacterial origin". As far as I understand, we still have a grey zone in the replicator space, which is of great interest for the generations of researchers. In a benchmark studies like that, it would be beneficial to take this into account and separately process the "unknown" category to ensure it does not bias the results in either direction. I did not explicitly find this mentioned in the manuscript, but it is possible that I may have overlooked it.

Minor remark: why using "Viruses of microbes" instead of "bacteriophages" - do you expect any archaeal viruses in your dataset?

**Authors Response**

**Point-by-point responses to the reviewers’ comments:**

*Reviewer 1*

*Major comments:*

*First, the datasets used for the evaluation are environmental samples, and thus of unknown composition. Accordingly, I don't think it is easy (not possile at all?) to say which tool performed better. A mock sample (of known composition) would be more useful for comparison purposes, I believe. The authors argue against the latter using a couple examples from the literature, but all these examples mentioned were small diversity mock samples, and thus limited, as the authors also suggest. A more appropriate dataset may be to sample 100 to 1000 viral genomes from the public databases, simulate a metagenome from them and then query the tool against these mock datasets while first removing the chosen genomes from the training datasets of the tools and/or the public database the tools are using (and possibly mixing them also with similar microbial datasets to increase complexity/challenge for the tools). Further, one can choose the genomes to represent different levels of novelty compared to the remaining genomes in the database/training dataset (same species; different species of the same family; different families etc.) in order to assess how the tools do with increasing levels of novelty of the target viral sequences.*

We respectfully disagree that a mock sample of known composition would be more useful for comparing tools in their ability to identify unknown viruses in metagenomes, because the tools were trained on that same data of known composition. We have explained this opinion in our manuscript (lines 86-87). Still, as multiple people have asked us this same question, we have now constructed a mock community with the viral (6,155 sequences) and bacterial genomes (22,552 sequences) deposited into the RefSeq database between 2020-01-12 and 2023-11-13. We chose 2020-01-12 because it is the date when the reference database for the last evaluated tool was downloaded (VirSorter2). We cut simulated contigs from the viral and bacterial genome sequences and ran all nine virus identification tools. The results showed a similar trend as with the real-world metagenomic data. To assess the sequence similarity between the viruses in the mock and reference data, we compared the viral genomes submitted after 2020-01-12 with previously deposited ones and used the similarity categories to interpret the results. As expected, machine-learning tools were better at identifying viruses in all similarity categories than homology-only tools, including viruses with low or high similarity to known ones. This new analysis is now described in the Methods and Results sections (lines 154-163, 434-444), including an additional figure (Supplementary Figure 10).

*Second, the comparison found that the machine-learning tools find more viral contigs. However, I believe the only data used to say that these contigs were indeed viral is that the sequences were found in the viral fraction (viromes). However, it is not certain that these sequences are indeed viral; in my view, they could represent anything such as virions, gene transfer agents, other mobile elements, plasmids, junk of viral preps, right? If there is no homology to any available isolate genome, how the authors could be certain that they are indeed viral (and specifically for the purpose of the study bacteriophages), and thus conclude that machine learning tools perform better? I would argue that plasmid and other mobile element sequences often look like phage genomes e.g., have repeats, integrases, lots of hypothetical genes. So, I would not be surprised if the machine learning tools identify as viral such (non-viral) sequences.*

We understand your concerns about the potential non-viral elements in the viral fraction. We had discussed this issue in the section “Comparison to other existing benchmarking work”, but now realize that the discussion is not in the appropriate section. Thus, we split this section in two and added a new section titled “The advantages and limitations of using real-world metagenomic data” (lines 497-542). In this section, we discuss potential “contamination” in both viral and microbial fractions and our efforts to address these issues. Besides, we changed the title of the section “Characterization and annotation of predicted viral contigs” to “Validation of predicted viral contigs in the real-world testing dataset” and clarified our validation results (lines 404-432):

We selected virome datasets which were obtained using DNase treatment beacause DNase treatment can remove free microbial DNA in the surrounding environment and improve virome quality effectively. The quality of the virome datasets were further assessed by ViromeQC (lines 502-507).

We removed the homologous contigs between viral and microbial fractions. Those homologous contigs present in both fractions could represent plasmids, prophages, and other elements shared between the two fractions (lines 514-516).

We used CAT and hmmsearch to characterize and annotate the real-world testing datasets. More viral signals were observed in the viral than in the microbial fraction. This is visible in the HMM profile heatmaps in Figure 5 as a stronger blue signal in the viral fraction than in the microbial fraction. We now clarify and discuss this in lines 407-424 and lines 508-510.

We acknowledge the limitations of our methods and mention that our real-world metagenomic approach is a complementary method to prior simulation studies (lines 520-525).

*Specific/minor comments:*

*Line 58. Viral genome integration too (prophages).*

Revised: “... and (4) the presence of prophages in microbial genomes.” (line 61-62)

*Line 124. No threshold on identity, e.g. >95% nt identity, or Minimap2 has a default sequence identity threshold?*

We used the default setting of Minimap2. Minimap2 does not have a specific identity cutoff but it is designed for mapping long reads with 5-15% mismatches, see https://github.com/lh3/minimap2/issues/424. We now clarify this in the Methods section (line 146).

*Methods section: The bioinformatics part looks robust and clear to me overall. But I am not sure about confirming/validating that whether or not a contig is indeed viral, the CheckV is the preferred approach instead, or in addition to, searching against the viral isolate genome database (~3 million genomes available).*

See our answers to comment 2 for the annotation and characterization of specific predicted sequences. We additionally used both CheckV viral and microbial hallmark gene HMM profiles to validate of the testing dataset using hmmsearch (see Methods in lines 203-204 and Fig. 5). In addition, we assessed the completeness of viral contigs usign the CheckV tool (see Methods in lines 189-190 and Supplementary Fig. 4).

For the second question about searching against the isolate viral genome database, we are unsure what database the Reviewer is thinking of. While there are 14M viruses in the NCBI Viruses database those are not isolates, and most are human-infecting RNA viruses. The remaining sequences are redundant. This is why we prefer to use the RefSeq database. We note that one of the tested tools performs the analysis suggested by the Reviewer. MetaPhinder uses a BLASTn search against the viral reference database and average nucleotide identity thresholds to classify viral contigs in metagenomes and identified <1% of the viruses in our virome dataset (Fig. 1). This clearly shows the added value of machine learning tools and our analysis.

*Lines 225-228. The major result of the study seems to be that CNN methods find more viral contigs. The only data in support of this appears to be that the sequences were found in the viral fraction but such sequences could be anything, right? Meaning if there is not enough homology to a genome, they could be virions, gene transfer agents, other mobile elements, plasmids, junk of viral preps, correct? Consistent with this assumption, the sequences CNN identified as viral that the homology did not find were marked as NA/not-identified by the later right, not microbial or other origin?*

*Line 260 and elsewhere. I think it would be useful for the authors to mention, in the main text, what caused the lower specificity of the machine learning tools. Is it that they identified microbial sequences as viral for example? How much/often?*

Please see our answers to comment 2. In addition, We added the measure we took to ensure the quality of the real-world testing datasets in the introduction for clarification (see lines 103-106).

Indeed, the Reviewer is right that the machine learning tools have a lower specificity, which is reflected in the sequences that were detected in the microbial fraction. Those sequences could be false positives, i.e. microbial sequences spuriously identified as viral, or also real viral sequences that were still present in the microbial fraction. This is now clarified in lines 272-278.

*Line 329-331. Have you searched these contigs for matches against plasmids or other mobile elements in the NCBI/UNIPROT databases? And, plasmid is just one of the possibilities if the query sequences are non-viral, not all possibilities.*

As already mentioned by the Reviewer, it is difficult to distinguish viruses from plasmids and other mobile genetic elements (MGEs). Van Dijk et al. predicted MGEs including phages, plasmids, IS-elements, ICEs, and integrons, and found considerable overlap between the categories. This does not necessarily mean that the tools perform poorly, but also suggests that recombination is rampant (https://www-nature-com.proxy.library.uu.nl/articles/s43705-023-00294-w #Fig3). We added a discussion of this in lines 508-510.

*Line 346. I would not call RefSeq sequence data "artificial" (they are real data). Maybe "test dataset of selected reference genome/sequences"?*

Revised: “Most tools performed significantly worse on the mock community dataset than on the simulated dataset derived from reference genomes…” (line 479-480)

*Reviewer 2*

*Major comments:*

*My main concern is the use of datasets from only three biomes - it calls into question the possible generalization of your findings.*

The three samples were selected to be as diverse as possible, see the recent analysis of global microbiomes here: https://www.nature.com/articles/s41559-023-02027-7 #Fig1d. Of course one can always keep adding more data, but we believe that these three biomes allow all virome researchers to obtain insights from our study. Our analysis critically depends on the availability of matched virome and microbiome datasets from the same samples, and such data is rare. We agree that samples from three individual studies cannot be assumed to represent all microbiomes. However, we saw that different tools have very similar performance in the different biomes, indicating that our results are generalizable. We modified the text in lines 100-102 and lines 526-542 to clarify this.

*Another potential issue is the potenital non-viral contigs in size-fractionated samles (virions, plasmids and other entities from the dark corners of virosphere), as well as possible viral fraction in bacterial samples*

Please see our answers to comment 2.

*Also, in a benchmarks like that I always curious about this binary opposition "viral/bacterial origin". As far as I understand, we still have a grey zone in the replicator space, which is of great interest for the generations of researchers. In a benchmark studies like that, it would be beneficial to take this into account and separately process the "unknown" category to ensure it does not bias the results in either direction. I did not explicitly find this mentioned in the manuscript, but it is possible that I may have overlooked it.*

This is a good point, which we have now further discussed and clarified in the text (e.g. see our answer to comment 7). The gray zone reflects the overlap between different MGE sequences. It is difficult to identify them but we are getting close to disentangling this issue. For example, PPR-Meta has three scores of viruses, plasmids, and chromosomes.

*Minor remark: why using "Viruses of microbes" instead of "bacteriophages" - do you expect any archaeal viruses in your dataset?*

We use "Viruses of microbes" instead of "bacteriophages" because the testing dataset might contain archaeal and eukaryotic viruses. In another study, we predicted hosts of the viruses in the tomato dataset and found that some of them infect archaea (data not shown).

**Second round of review**

**Reviewer 1**

The revised article by Wu et al., represents a much-improved version compared to the first submission. The authors have addressed all my major concerns and those of other reviewers, including the suggestion to include a couple mock datasets of substantial complexity for testing. I felt the authors did a good job overall at integrating reviewers' input and revising the manuscript. I have no major comments remaining. Yes, in my previous email I was referring that a database like the viral part of RefSeq would be appropriate; apologies to the authors for not being clear enough about this point.
